# Estimation of vaccination coverage and associated factors in older Mexican adults

**DOI:** 10.1017/S0950268823001218

**Published:** 2023-08-14

**Authors:** Héctor García-Hernández, Judith Zárate-Ramírez, Ashuin Kammar-García, Carmen García-Peña

**Affiliations:** 1 Researcher, Health Research Division, National Institute of Geriatrics, Mexico City, Mexico; 2 Research Fellow, Health Reserch Division, National Institute of Geriatrics, Mexico City, Mexico; 3 General Director, National Institute of Geriatrics, Mexico City, Mexico

**Keywords:** health system, immunization, elders, vaccines, influenza, pneumococcus, tetanus, diphtheria

## Abstract

National vaccination programmes recommend the influenza vaccine for older adults, but this population group has the greatest morbidity and mortality from other preventable vaccine diseases. The aim of this article is to estimate the vaccine coverage in adults aged 65 years and older and to analyse the factors that could increase or decrease vaccination uptake probability for the three listed vaccines in the national vaccination programme (influenza, tetanus and diphtheria, and pneumococcus) and the full scheme in Mexico. We conducted an analytical cross-sectional study with 2012, 2018, and 2021 rounds from the National Health and Nutrition Survey, in which we calculated the vaccine coverage estimations and performed multivariable logistic regression models to analyse the factors related to vaccine uptake. Tetanus and diphtheria vaccines had the greatest coverage estimation in all years (59–71%), whereas the pneumococcus vaccine had the lowest (32–53%). Full scheme vaccine coverage decreased from 37.80% to 24.77% in 2012 and 2021, respectively. The National Health Card property, morbidity, being a beneficiary of any health system institution, and use of preventive services increased the probability of vaccine uptake. In conclusion, vaccine coverage in older Mexican adults decreased over time, and the Mexican health system plays a strategic role in immunisation.

## Introduction

Vaccines are one of the most successful public health strategies. Mass immunisation programmes in previous decades have eradicated smallpox throughout the world and poliomyelitis in 190 countries [1, 2]. Additionally, between 2010 and 2018, measles vaccinations prevented 23 million deaths worldwide, and the mortality rate of children under 5 years of age decreased by 24% [3].

Among older adults, the World Health Organization (WHO) only recommends influenza vaccines as routine for this age group, despite it being well established that vaccines contribute to promoting healthy ageing and there is scientific evidence about the protection provided by other vaccines, like pneumococcus [4–7]. Moreover, vaccine uptake strategies, mostly focused on early ages, had reduced the morbidity and mortality of preventable vaccine diseases (PVDs) in children [1, 5]. Also, since demographic transition has increased the mean age of the population that may be susceptible to PVD, the prevalence of such diseases has risen in older adults contrary to what happens in infants [8]. Moreover, 90% of deaths caused by the influenza virus occur in elderly individuals [9], and 80% of acute respiratory infection cases require hospitalisation in that age group [10].

In Mexico, mortality of PVD has been on a downward trend since the end of the last century for children and older people [11]; pneumonia and influenza mortality in the age group of 1–4 years went from 90 deaths per 100,000 births in 1980 to 6.5 in 2009. However, this trend has been less marked in older adults aged 65 years (411.2 deaths per 100,000 habitants in 1980 to 141.2 in 2009) [12]. As a consequence, mortality of PVD has been concentrated in older adults since the beginning of this century (in 2020, pneumonia mortality was 30 times higher in persons aged 65 years and older than in children under 5 years [303 and 10 deaths per 100,000 habitants], this situation was also observed with influenza [1.724 versus 0.189 deaths per 100,000 habitants]) [13]. These data emphasise that vaccination needs to be understood across the life cycle [3] and not solely in the first years of life.

Furthermore, even with the importance of vaccines and all of their potential benefits, vaccine coverage (VC) still has an important gap in reaching 100% of the older population. In the United States, tetanus, influenza, and pneumococcus VC were below 65%, 75%, and 65%, respectively, in adults over 65 years in 2015 [10]. In Bogotá, Colombia, vaccine percentages for the same pathogens were similar [9], while in Brazil, influenza VC was 73% among individuals over 60 years in 2015 [14].

In Mexico, the national vaccine programme includes three different vaccines for older people: pneumococcus, influenza, and tetanus and diphtheria (Td), which are specified in the National Health Card (NHC). The NHC is a strategic document to register all activities of disease prevention and health promotion by age group, which are mandatory across the whole Mexican health system. The first vaccine is indicated in persons over 65 years in a single dose, and in people between 60 years and 64 years who have risk factors such as chronic diseases, alcoholism, cirrhosis, asplenia, overweightness, immunosuppression, and cerebrospinal fluid fistulae, and people living in asylums. The second vaccine is recommended annually to all elderly individuals over 60 years. One dose of Td vaccine is indicated for every 10 years in persons over 60 years [15]. Furthermore, available information about percentage coverages exhibits a range from 62% to 69% for Td, 51% to 64% for influenza, and 44% to 62% for pneumococcus in 2012 [16].

Additionally, VC is influenced by multiple factors that may promote or hinder its coverage. In Brazil, Sato et al. [14] demonstrated that persons aged 70–79 years, individuals with access to health system programs, and persons with two or more chronic diseases promote the chances of vaccination. Meanwhile, similar results have been found in Colombia [9]. Moreover, in China, health literacy and vaccination awareness increased the probability of influenza vaccination [17].

However, there is no information on potential factors that could increase or decrease vaccination probability in Mexico for the vaccines indicated to older adults in the national vaccine programme. Consequently, the main purpose of this study was to estimate the VC in adults aged 65 years and older and to analyse the factors related to VC for the three vaccines listed in the national vaccine programme, using the 2012, 2018, and 2021 rounds from the National Health and Nutrition Survey (Encuesta Nacional de Salud y Nutrición, ENSANUT).

## Methods

### Study design

We conducted an analytical cross-sectional study on residents of Mexico who were surveyed in the 2012, 2018, and 2021 rounds from the National Health and Nutrition Survey. The three rounds of the ENSANUT used a probabilistic, two-stage, stratified, and conglomerate sampling of households with a national and urban/rural representation, and the survey is also representative at the state level (32 states) for the 2012 and 2018 rounds, and at the regional level (9 regions) for the 2021 round. Its main objective is to determine specific relevant indicators of health and nutrition conditions in Mexico. Additional information about each survey in chronological order can be found elsewhere [18]. All of the rounds were approved by the National Institute of Public Health Research Ethics Committee, wherein all of the participants signed an informed consent letter.

### Participants

We included adults aged 65 years or older who completed the immunisation survey section. The exclusion criteria included persons not answering the age or sex questions. Therefore, the sample size in the three ENSANUT rounds was 194,923 in 2012, 158,044 in 2018, and 13,402 in 2021, from which 13,869, 13,922, and 2,025 individuals, respectively, corresponded to adults aged 65 years or older who answered the previously mentioned questions.

### Analysed variables

Vaccination coverage was defined as the presence of administration of each of the vaccines listed in the national vaccine programme, regardless of the recommendation dates stated in the NHC. For the pneumococcus and Td vaccines, we considered persons over 65 years who were vaccinated with a single dose. For influenza coverage, we considered those who were vaccinated in the year before the survey. Consequently, we define a full immunisation scheme if the older adult had received the three vaccines. All the data during each of the surveys were obtained in an interview with every subject. In this interview, the interviewer first asked for the NHC; if the participant presented the document, the information about vaccination coverage was obtained from it. However, if the subject did not have the NHC, the interviewer inquired about the vaccines that were taken by self-reporting.

The sociodemographic characteristics that were included in the study were age (categorised as 65–74, 75–84, and equal to or more than 85 years), sex, marital status (categorised as: married or in union, and single [in which the divorced, separated, and widowed subjects were included]), indigenous language speaker, education (categorised as below elementary school, completed elementary school, completed middle-high school, and above middle-high school), literacy (the ability to read and write), beneficiary of any social programmes (which included all programmes related to scholarships, conditional cash transfers, governmental pensions, and in-kind food support), work status (defined as having had a paid job in the last week), and type of residency (urban [≥2,500 inhabitants] or rural [≤2,500 inhabitants]).

We also included health-related variables like NHC property, being a beneficiary of any health system institution (regardless of whether the institution belongs to social security or not), the use of health care preventive services (pap smear, papillomavirus screening, breast examination, mammography, tuberculosis screening, overweight and obesity screening, high cholesterol and triglycerides screening, diabetes mellitus and hypertension screening, prostate cancer screening, and digital rectal examination), morbidity (defined as one or more previously diagnosed diseases [hypertension, diabetes mellitus, cardiovascular disease, stroke, hypertriglyceridemia, and hypercholesterolemia] made by a medical physician), and presence of depression symptoms (defined as a score more or equal to 5 of the Center for Epidemiological Studies abbreviate scale [CESD-7], validated to the Mexican population) [19].

### Statistical analysis

We estimated VC for every vaccine and for the full scheme. The results were analysed to reflect the entire population by using population projection data. The estimations were presented as percentages, confidence intervals at 95% (95% CI), and coefficients of variation (CV). The CV is a quality measure to evaluate survey estimations with a probabilistic sample; its interpretation depends on certain limits of acceptance to determine the quality of the estimation made for surveys that involved dwellings, households, or units other than economic, and a value of 0–15, 16–30, and more than 30 represent a high, moderate, and low-quality level of the estimation, respectively [20].

Subsequently, we performed a descriptive analysis of sociodemographic and health characteristics with the expanded data (which is presented as a percentage and 95% CI for each variable) by all years of each survey.

Different multivariable logistic regression models were performed by using the stepwise forward method (considering *P* < 0.1 as a significant contribution to the model of each variable) to determine the factors related to VC in the full scheme and for each vaccine. Subsequently, variables selected via the stepwise forward method were used to perform other multivariable logistic regression models that included variables that the authors determined as being important (sex, age categorised, education, and type of residency) by using the Enter method [21]. The results for every model are presented as odds ratios (ORs) and 95% CIs.

A value of *P* < 0.05 was considered as statistical significance, and all the analyses were performed by adjusting the results for complex survey design with the SVY command in the STATA v.14 software.

## Results

We included 13,869, 13,922, and 2,025 observations from adults aged 65 years or older who answered the age and sex questions in 2012, 2018, and 2021, respectively. The mean age for each year was 73.99 (standard deviation [SD]: 7.17), 73.88 (SD: 7.31), and 73.00 (SD: 6.62). From the 13,869, 13,922, and 2,025 observations, 4,051, 3,912, and 1,137 presented the NHC in 2012, 2018, and 2021.

Table 1 presents the VC estimation for every vaccine and the full scheme for the total population of 65 years or older by sex. The pneumococcal vaccine had the lowest value in all years. Although the Td vaccine had the highest percentage for all of the time periods, in 2021 the percentage was 61.24%, in contrast with 55.64% for influenza and 36.30% for pneumococcus coverage in the same year. Additionally, in 2021, 24.77% of Mexicans aged 65 years or older had the full immunisation scheme. VC percentages also decreased over time. Moreover, 2012 was the year that demonstrated the highest VC values. However, 2018 had the most significant percentage decline, reaching 20.41% for full scheme vaccinations. Furthermore, women had higher VC percentages than men for all years and vaccine types. In parentheses are the CV numbers. Their values were lower than 15; thus, we can assume that the estimations had a high level of quality.

**Table 1.** Vaccination coverage in adults aged 65 years or older, for influenza, pneumococcus, tetanus/diphtheria, and full scheme for 2012, 2018, and 2020

Year	2012			2018			2021		
Vaccine coverage	Total (*n* = 7,524,148)	Men(*n* = 3,508,521)	Woman(*n* = 4,015,628)	Total(*n* = 11,400,388)	Men(*n* = 4,983,949)	Woman(*n* = 6,416,439)	Total(*n* = 10,941,860)	Men(*n* = 5,792,980)	Woman(*n* = 5,148,880)
Full scheme	37.80% (3.02) 35.58–40.07	35.55% (4.34) 32.58–38.63	39.67% (3.73) 36.89–42.71	20.41% (3.69) 18.97–21.92	19.43% (5.87) 17.29–21.77	21.16% (4.56) 19.33–23.12	24.77% (6.71) 21.64–28.19	23.57% (9.71) 19.36–28.38	26.11% (8.86) 21.81–30.92
Influenza	59.50% (2.01) 57.12–61.83	57.60% (2.73) 54.48.60.65	61.16% (2.60) 57.99–64.24	46.33% (2.09) 44.44–48.24	45.02% (3.17) 42.24–47.85	47.35% (2.86) 44.71–50.02	55.64% (3.01) 52.31–58.90	54.41% (4.42) 49.63–59.09	57.02% (4.03) 52.44–61.48
Pneumococcus	52.53% (2.18) 50.27–54.78	50.01% (3.49) 46.59–53.43	54.73% (2.67) 51.85–57.58	36.51% (2.54) 34.70–38.35	35.72% (3.81) 33.10–38.44	37.11% (3.43) 34.64–39.64	36.30% (4.67) 33.03–39.70	32.86% (8.01) 27.89–38.24	40.17% (5.43) 35.95–44.53
Tetanus/diphtheria	69.78% (1.47) 67.72–71.76	68.32% (2.14) 65.38–71.12	71.06% (1.93) 68.28–73.68	60.43% (1.79) 58.33–62.50	59.95% (2.54) 56.93–62.90	60.81% (2.25) 58.08–63.46	61.24% (3.09) 57.45–64.90	60.56% (4.61) 54.93–65.90	62.02% (3.81) 57.26–66.55

Note: Coefficient of variation is in parenthesis. Confidence intervals (CI) at 95% are found at the end of every cell.

The descriptive analysis of the sociodemographic and clinical characteristics for each variable is presented in Table 2 for the years 2012, 2018, and 2021. We observed that the oldest persons represented a lower percentage of VC in all years. Additionally, the percentage values of women were higher than the values for men for almost all years and vaccines. Moreover, there were more respondents with NHC and beneficiaries of any health system institution among vaccinated older adults. We also observed that among vaccinated adults aged 65 years or older, few observations had completed middle-high school and above middle-high school. There was also an increase in full vaccination coverage in literate older adults (70.86% in 2012 and 85.80% in 2021), and a decrease in indigenous language speakers (10.64% in 2012 and 5.56% in 2021). Furthermore, the use of preventative services in vaccinated adults aged 65 years or older decreased from 76–81% in 2012 to 27–30% in 2021, depending on vaccine type. Unexpectedly, more than half of the adults aged 65 years or older had depression symptoms in almost every vaccinated group. Finally, sick older adults were more vaccinated than the healthier ones, and percentages were higher in older adults with urban residency. Percentages for adults aged 65 years or older who were married or in union and those who worked remained constant in every survey.

**Table 2.** Descriptive analysis for vaccinated adults aged 65 years or older, in 2012, 2018 and 2021

Year	2012 (*N* = 7,524,148 older adults)				2018 (*N* = 11,400,388 older adults)				2021 (*N* = 10,941,860 older adults)			
Variable	Complete scheme (*n* = 4,062,981)	Influenza (*n* = 6,394,864)	Pneumococcus (*n* = 5,646,086)	Tetanus/difteria (*n* = 7,499,921)	Complete scheme (*n* = 2,326,591)	Influenza (*n* = 5,282,712)	Pneumococcus (*n* = 4,161,826)	Tetanus/difteria (*n* = 6,890,167)	Complete scheme (*n* = 2,710,627)	Influenza (*n* = 6,087,723)	Pneumococcus (*n* = 3,972,005)	Tetanus/difteria (*n* = 6,701,342)
Age												
65–74	60.14% (57.10–63.11)	58.76% (56.02–61.46)	58.69% (55.67–61.66)	60.48% (57.77–63.13)	61.08% (57.25–64.78)	64.49% (61.88–67.02)	59.59% (56.65–62.46)	64.07% (61.73–66.35)	62.72% (55.47–69.44)	62.12% (57.43–66.59)	62.41% (57.01–67.51)	65.83% (61.88–69.57)
75–84	31.76% (28.87–34.79)	32.61% (30.13–35.20)	32.67% (29.87–35.59)	31.34% (28.79–34.00)	31.33% (27.79–35.09)	27.55% (52.91–29.93)	31.96% (29.21–34.84)	28.07% (25.99–30.23)	32.05% (24.71–40.41)	31.05% (26.49–36.00)	31.50% (25.90–37.68)	29.34% (25.68–33.28)
> = 85	8.09% (6.52–10.00)	8.62% (7.27–10.19)	8.63% (7.15–10.39)	8.18% (6.81–9.79)	7.59% (5.84–9.82)	7.96% (6.56–9.63)	8.45% (6.94–10.25)	7.86% (6.61–9.32)	5.23% (27.44–9.72)	6.84% (4.47–10.32)	6.10% (3.94–9.33)	4.84% (3.24–7.17)
Sex												
Men	43.85% (40.66–47.10)	45.14% (42.45–47.85)	44.39% (41.48–47.34)	45.66% (43.11–48.26)	41.63% (37.96–45.41)	42.48% (39.76–45.23)	42.79% (39.87–45.75)	43.37% (41.03–45.74)	50.39% (43.43–57.32)	51.77% (47.85–55.67)	47.93% (42.29–53.62)	52.35% (48.47–56.19)
Woman	56.14% (52.90–59.34)	54.86% (52.15–57.54)	55.61% (52.66–58.52)	54.34% (51.77–56.89)	58.37% (54.59–62.05)	57.52% (54.76–60.24)	57.21% (54.25–60.13)	56.63% (54.26–58.97)	49.61% (42.67–56.56)	48.23% (44.33–52.15)	52.07% (46.38–57.71)	47.65% (43.81–51.53)
Married or in union	55.81% (52.46–59.10)	55.45% (52.79–58.09)	56.09% (53.10–59.02)	56.80% (54.1–59.41)	57.51% (53.84–61.10)	57.22% (54.46–59.93)	56.18% (53.32–59.01)	57.41% (55.07–59.72)	66.04% (58.74–72.64)	60.98% (56.51–65.27)	62.50% (56.51–68.11)	61.34% (56.98–65.53)
Have National Health Card	78.23% (75.22–80.96)	73.39% (70.83–75.81)	74.91% (72.30–77.35)	70.36% (67.91–72.69)	85.07% (82.23–87.53)	70.67% (67.64–73.52)	80.46% (77.80–82.88)	68.06% (65.62–70.40)	73.64% (67.58–78.93)	62.15% (57.81–66.30)	71.24% (66.12–75.87)	64.88% (60.90–68.67)
Affiliated with health system	88.55% (86.27–90.45)	86.91% (85.02–88.59)	87.06% (84.88–88.97)	85.83% (83.74–87.69)	96.01% (94.47–97.13)	92.95% (90.89–94.58)	94.66% (93.11–95.87)	92.39% (91.09–93.51)	83.79% (78.50–87.97)	77.19% (73.01–80.90)	81.51% (76.76–85.48)	77.26% (73.61–80.53)
Indigenous language speaker	10.64% (8.75–12.89)	9.13% (7.56–10.99)	9.54% (7.94–11.43)	8.80% (7.29–10.59)	14.36% (11.51–17.78)	9.89% (8.26–11.81)	12.54% (10.47–14.94)	9.80% (8.28–11.56)	5.56% (2.98–10.13)	4.47% (2.86–6.92)	6.70% (3.92–11.23)	5.67% (3.44–9.19)
Education												
Below elementary school	29.62% (26.75–32.67)	29.22% (26.73–31.84)	30.30% (27.68–33.05)	29.64% (27.26–32.14)	25.33% (22.35–28.56)	22.26% (20.19–24.46)	25.02% (22.66–27.53)	21.70% (19.96–23.54)	15.37% (11.20–20.72)	17.74% (14.36–21.71)	17.29% (13.78–21.47)	18.19% (15.16–21.68)
Complete elementary school	57.13% (53.72–60.48)	54.91% (52.07–57.72)	55.52% (52.41–58.59)	54.66% (51.88–57.41)	52.99% (49.17–56.77)	49.57% (46.69–52.44)	51.03% (48.01–54.05)	51.08% (48.68–53.46)	55.06% (47.94–61.98)	51.76% (46.56–56.93)	54.47% (48.99–59.84)	51.31% (46.37–56.22)
Complete middle-high school	6.26% (4.44–8.75)	6.35% (4.94–8.19)	5.95% (4.58–7.70)	5.95% (4.74–7.45)	9.33% (7.46–11.61)	10.97% (9.19–13.06)	9.96% (8.19–12.06)	10.71% (9.32–12.27)	16.41% (11.74–22.46)	13.10% (10.02–16.94)	13.91% (10.28–18.56)	13.80% (11.02–17.14)
Above middle-high school	6.97% (5.51–8.78)	9.52% (7.87–11.46)	8.21% (6.67–10.06)	9.73% (8.09–11.67)	12.34% (9.80–15.43)	17.19% (14.92–19.72)	13.98% (11.81–16.46)	16.50% (14.62–18.57)	13.15% (8.08–20.68)	17.39% (12.32–23.96)	14.31% (10.00–20.07)	16.69% (12.62–21.74)
Literacy	70.86% (67.91–73.64)	72.98% (70.44–75.38)	71.15% (68.49–73.67)	73.34% (70.91–75.64)	67.39% (63.88–70.72)	72.02% (69.56–74.35)	68.16% (65.49–70.72)	72.35% (70.29–74.31)	85.80% (81.14–89.46)	87.91% (84.95–90.35)	84.93% (81.08–88.11)	86.17% (83.38–88.57)
Beneficiary of social programs	45.82% (42.45–49.24)	44.49% (41.51–47.50)	45.06% (41.92–48.23)	42.73% (40.03–45.48)	63.27% (59.52–66.87)	55.32% (52.43–58.17)	59.93% (56.97–62.83)	53.46% (50.97–55.93)	69.41% (62.14–75.84)	65.45% (60.40–70.17)	69.43% (63.85–74.49)	64.01% (59.35–68.41)
Use preventive services	80.46% (77.94–82.75)	78.05% (75.85–80.10)	78.74% (76.44–80.87)	76.35% (74.10–78.47)	53.31% (49.51–57.07)	47.43% (44.63–50.26)	50.28% (47.34–53.22)	47.48% (45.11–49.86)	27.38% (21.78–33.80)	29.42% (25.91–33.20)	27.71% (23.18–32.75)	27.50% (23.89–31.43)
Work status	18.43% (15.94–21.21)	18.69% (16.70–20.86)	18.96% (16.95–21.15)	19.97% (18.12–21.96)	21.26% (18.53–24.29)	22.89% (20.75–25.19)	22.27% (20.09–24.62)	25.25% (23.37–27.24)	21.79% (17.19–27.22)	19.96% (17.03–23.25)	19.90% (15.99–24.48)	22.83% (19.58–26.45)
With depression symptoms	52.70% (49.12–56.25)	51.38% (48.62–54.14)	51.73% (48.78–54.67)	50.44% (47.83–53.04)	60.10% (56.34–63.75)	59.99% (57.25–62.67)	59.35% (56.48–62.16)	58.59% (56.23–60.91)	55.46% (47.15–63.48)	54.17% (49.14–59.11)	57.38% (51.12–63.41)	54.85% (50.13–59.50)
Morbidity	66.56% (63.57–69.42)	65.75% (63.34–68.08)	65.05% (62.39–67.62)	64.43% (61.98–66.81)	71.80% (68.31–75.05)	69.29% (66.62–71.84)	71.04% (68.30–73.64)	67.95% (65.64–70.18)	67.50% (60.92–73.81)	64.43% (60.64–68.05)	66.63% (61.08–71.76)	62.14% (58.05–66.07)
Type of residency												
Urban	72.21% (68.58–75.56)	74.17% (71.22–76.92)	73.95% (70.83–76.84)	75.72% (73.02–78.23)	69.56% (66.16–72.76)	74.97% (72.83–76.99)	72.98% (70.66–75.19)	75.86% (74.06–77.58)	75.09% (66.79–81.88)	78.87% (74.10–82.97)	76.83% (70.23–82.34)	76.79% (72.18–80.84)
Rural	27.79% (24.44–31.41)	25.83% (23.08–28.78)	26.05% (23.16–29.17)	24.28% (21.77–26.98)	30.44% (27.24–33.84)	25.03% (23.00–27.17)	27.01% (24.81–29.34)	24.14% (22.42–25.94)	24.91% (18.13–33.21)	21.13% (17.03–25.90)	23.17% (17.66–29.78)	23.21% (19.16–27.81)

Note: Confidence intervals (CI) at 95% are found at the end of every cell.

Finally, multivariable logistic regression models are shown in Table 3. The blank spaces represent the variables that were excluded via the stepwise forward method. The only variables with statistically significant values in all years and vaccine types were NHC (although the size of the effect decreased over time) and morbidity (the size of the effect stayed constant over time). For the NHC property, in 2012, the model of the full scheme demonstrated that this variable increased more than three times the probability to be vaccinated (OR: 3.575, 95% CI: 3.16–4.03), in contrast with more than two times’ probability (OR: 2.357, 95% CI: 1.87–2.96) in 2021. For morbidity, the odds ratio for the full scheme were 1.230 (95% CI: 1.09–1.37) in 2012 and 1.372 (95% CI: 1.09–1.73) in 2021. It is important to mention that although the probabilities decreased over time, both variables were always observed to be factors positively associated with vaccination; however, other variables such as age categories, sex, married or in union status, speaking an indigenous language, education, literacy, work status, depression symptoms, and urban residency did not demonstrate clear, constant, and significant relationships.

**Table 3.** Logistic regression models (OR) for every vaccine indicated in the NHC for adults aged 65 years or older

	2012 (*n* = 6,379)				2018 (*n* = 6,410)				2021 (*n* = 2,023)			
Variable	Complete scheme	Influenza	Pneumococcus	Tetanus/diphtheria	Complete scheme	Influenza	Pneumococcus	Tetanus/diphtheria	Complete scheme	Influenza	Pneumococcus	Tetanus/diphtheria
Age												
65–74	Reference	Reference	Reference	Reference	Reference	Reference	Reference	Reference	Reference	Reference	Reference	Reference
75–84	0.910 (0.80–1.02)	0.994 (0.87–1.12)	1.017 (0.90–1.14)	0.836* (0.73–0.95)	0.933 (0.81–1.07)	0.892 (0.79–1.00)	1.034 (0.91–1.17)	0.838* (0.74–0.94)	1.051 (0.81–1.35)	1.097 (0.89–1.35)	1.005 (0.81–1.26)	0.825 (0.66–1.02)
> = 85	0.810* (0.66–0.98)	0.845 (0.69–1.02)	0.902 (0.75–1.09)	0.600* (0.49–0.73)	0.781* (0.61–0.99)	0.849 (0.70–1.02)	0.825 (0.67–1.01)	0.627* (0.52–0.76)	0.757 (0.47–1.21)	0.880 (0.61–1.26)	0.902 (0.61–1.33)	0.539* (0.37–0.78)
Sex												
Woman	1.018 (0.90–1.14)	1.106 (0.98–1.24)	0.991 (0.88–1.11)	0.916 (0.80–1.04)	1.074 (0.94–1.22)	1.076 (0.96–1.20)	1.101 (0.98–1.23)	0.971 (0.86–1.09)	1.165 (0.93–1.47)	1.142 (0.94–1.37)	1.217 (0.99–1.48)	1.049 (0.85–1.28)
Married or in union	–	–	–	1.051 (0.92–1.18)	–	1.118* (1.00–1.24)	–	1.164* (1.03–1.30)	1.233 (0.98–1.54)	–	–	1.151 (0.94–1.39)
Have National Health Card	3.575* (3.16–4.03)	3.432* (3.06–3.84)	3.628* (3.24–4.06)	3.504* (3.11–3.94)	4.187* (3.56–4.91)	2.324* (2.08–2.60)	4.263* (3.76–4.82)	2.421* (2.16–2.70)	2.357* (1.87–2.96)	1.732* (1.44–2.07)	2.433* (1.99–2.96)	2.228* (1.85–2.68)
Affiliated with health system	1.350* (1.14–1.59)	1.269* (1.08–1.47)	1.376* (1.17–1.60)	1.346* (1.15–1.57)	1.513* (1.15–1.98)	1.671* (1.38–2.01)	1.441* (1.16–1.78)	1.376* (1.15–1.63)	1.695* (1.29–2.23)	–	1.543* (1.22–1.94)	–
Indigenous language speaker	–	1.386* (1.15–1.66)	1.304* (1.17–1.60)	–	1.690* (1.38–2.07)	1.234* (1.02–1.48)	1.583* (1.30–1.92)	–	–	–	–	–
Education												
Below elementary school	Reference	Reference	Reference	Reference	Reference	Reference	Reference	Reference	Reference	Reference	Reference	Reference
Complete elementary school	1.074 (0.91–1.26)	1.068 (0.94–1.21)	1.122 (0.94–1.31)	1.068 (0.94–1.21)	0.990 (0.81–1.21)	0.970 (0.85–1.11)	0.901 (0.75–1.07)	0.936 (0.81–1.07)	1.236 (0.87–1.73)	1.168 (0.92–1.47)	1.192 (0.88–1.61)	1.066 (0.84–1.35)
Complete middle-high school	1.036 (0.77–1.38)	0.929 (0.71–1.21)	1.181 (0.88–1.57)	1.032 (0.77–1.37)	1.121 (0.84–1.49)	1.032 (0.84–1.27)	0.955 (0.74–1.23)	1.102 (0.88–1.36)	1.708* (1.09–2.67)	1.211 (0.86–1.70)	1.283 (0.86–1.92)	1.298 (0.91–1.84)
Above middle-high school	0.689* (0.53–0.88)	0.919 (0.74–1.14)	0.792 (0.62–1.00)	0.714* (0.57–0.89)	0.670* (0.50–0.89)	0.789* (0.66–0.96)	0.710* (0.55–0.91)	0.662* (0.55–0.79)	0.885 (0.55–1.42)	1.016 (0.73–1.41)	0.781 (0.52–1.17)	0.866 (0.62–1.21)
Literacy	0.811* (0.69–0.96)	–	0.785* (0.66–0.92)	–	0.878 (0.72–1.06)	–	0.877 (0.73–1.04)	–	0.668* (0.47–0.95)	–	0.659* (0.48–0.90)	–
Beneficiary of social programmes	1.128* (1.00–1.27)	1.166* (1.03–1.32)	1.112 (0.98–1.25)	1.123 (0.98–1.28)	1.212* (1.05–1.39)	1.204* (1.07–1.35)	1.188* (1.05–1.34)	–	1.294* (1.01–1.65)	–	1.289* (1.03–1.59)	–
Use preventive services	1.694* (1.49–1.92)	1.811* (1.59–2.05)	1.679* (1.48–1.90)	1.688* (1.48–1.92)	1.823* (1.60–2.07)	1.591* (1.43–1.77)	1.668* (1.49–1.86)	1.783* (1.59–1.99)	–	1.445* (1.16–1.79)	–	–
Work status	1.005 (0.87–1.15)	–	–	–	–	–	–	1.055 (0.92–1.20)	–	–	–	1.239 (0.97–1.58)
With depression symptoms	–	–	–	–	–	1.098 (0.98–1.22)	–	–	–	–	–	–
Morbidity	1.230* (1.09–1.37)	1.304* (1.16–1.46)	1.192* (1.06–1.33)	1.204* (1.07–1.36)	1.296 (1.12–1.49)	1.235* (1.10–1.38)	1.371* (1.21–1.54)	1.160* (1.03–1.30)	1.372* (1.09–1.73)	1.296* (1.07–1.57)	1.292* (1.06–1.58)	1.311* (1.08–1.59)
Urban residency	0.826* (0.73–0.93)	0.832* (0.73–0.94)	0.854* (0.75–0.96)	0.902 (0.79–1.03)	0.772* (0.67–0.88)	0.827* (0.73–0.93)	0.859* (0.75–0.97)	0.822* (0.72–0.92)	0.807 (0.63–1.04)	0.939 (0.75–1.16)	1.032 (0.82–1.29)	0.818 (0.65–1.02)

*Note*: Blank spaces are the variables that were taken out by the stepwise forward method.

Abbreviation: OR, odds ratio.

* *P* < 0.05.

Moreover, other variables that were positively associated with VC in almost all years were being a beneficiary of the health system and the use of preventative services. Therefore, those with health insurance increased their probability of having the full scheme by 35.0%, 51.3%, and 69.5% for 2012, 2018, and 2021, respectively. Adults aged 65 years or older who used preventative health services also increased their probability by 69.4% and 82.3% in 2012 and 2018.

## Discussion

The main purpose of this study was to determine the estimations of VC in older adults and to analyse the factors related to them for the three vaccines that are listed in the national vaccine programme. We found that VC in older Mexican people decreased over time. In 2012, 37.80% of adults aged 65 years or older had the full immunisation scheme; however, in 2021, this value was 24.77%. Furthermore, the main factors that increased VC probability were NHC property, morbidity, being a beneficiary of any health system institution, and the use of preventative health services.

Moreover, a reduction in coverage could be generated due to the COVID-19 outbreak. This sanitary emergency stopped health services such as immunisation programmes [22]. Consequently, VC decreased for almost all of the vaccines [23], which increased the risk of more PVD cases [22]. However, these low estimations are similar to other results from the USA (VC of 65%, 75%, and 65% for tetanus, influenza, and pneumococcus vaccine for adults older than 65 years in 2015, respectively) [10], Colombia (48.6%, 73%, and 57.8% for adults older than 60 years in 2015) [9], Brazil (73% for influenza among individuals older than 60 years in 2015) [14], and Italy (53% for influenza in the 2018–2019 season) [6] prior to the COVID-19 outbreak. These estimations certainly reflect weak vaccination programmes and few efforts to reach full immunisation schemes in older adults.

On the other hand, the Td vaccine had a higher vaccine estimation percentage than the pneumococcus and influenza vaccines. This effect may be related to the year of introduction of each vaccine. In Mexico, Pneumococcus, Td, and influenza vaccines were introduced in 1993, 1997, and 2004, respectively. Although pneumococcus vaccine was the first of the three to arrive in Mexico, this vaccine was not included in the national vaccine programme until 2006 by the Ministry of Health and was universally applied to adults over 65 years [24, 25]. Therefore, Td has been applied for a longer time. Meanwhile, influenza mostly depends on the publicity of the vaccine campaign each year.

To understand the identified factors related to VC, we must review the 5As taxonomy for vaccine uptake. This is a classification system that was developed to identify coverage gaps and vaccine uptake determinants. It has proposed five dimensions that influence vaccination: access, affordability, awareness, acceptance, and activation [26]. Our study demonstrated evidence that factors from access, affordability, and activation dimensions promote vaccine uptake in older Mexican adults.

Access is defined as the ability of individuals to be reached by or to reach recommended vaccines, including contact with health system services. In contrast, affordability is the ability of persons to afford vaccination at financial and nonfinancial costs [26]. In Mexico, health policy considers the vaccines included in the national vaccine programme as free of charge, regardless of whether persons are affiliated or not with any health system institution [15]. This may explain why using preventative health services, patients with morbidity, and being a beneficiary of any health system institution has an important effect on our regression analysis. Therefore, the Mexican health system plays an important role, despite its institutional segmentation depending on social security affiliation. Historically, only formal workers and their families could have access to social security health services [27] that have more resources [28]. This segmentation is relevant because it generates differences in mortality for certain diseases among both populations [29, 30].

However, being in touch in any way with the health system promotes vaccination uptake. Other investigations have found that health system affiliation increases vaccine coverage in Brazil (for influenza; OR: 1.36, *P* < 0.01, 95% CI: 1.10–1.69) [14], Colombia (for influenza; OR: 3.47, *P* < 0.001, 95% CI: 1.65–7.32, for pneumococcus; OR: 4.84, *P* < 0.001, 95% CI: 2.18–10.74, and for tetanus; OR: 4.55, *P* < 0.001, 95% CI: 2.11–9.83) [9], and the USA (for influenza; OR: 1.80, 95% CI: 1.46–2.21, and for pneumococcus; OR: 2.37, 95% CI: 1.95–2.88) [31]. This scenario may explain why women have higher VC estimates for all years and vaccine types; specifically, women have more effective access than men in Mexico [32].

There is also evidence that a free-of-charge vaccine policy increases vaccine probability. A previous study in China showed that the gratuity of vaccines increases the probability of a profit vaccine policy by 27.29 times (p < 0.001, 95% CI: 18.69–39.82) [17].

Consequently, reducing access or affiliation with health system institutions will negatively impact VC. Therefore, health systems must face specific challenges to facilitate VC, such as territorial access limitations, institutional affiliation restrictions, financial constraints, low promotion practices, sufficient vaccine stock [4], and lack of support for preventive health actions.

Moreover, the activation dimension is defined as the activities, prompts, and reminders that actively engage and incentivise persons towards vaccination uptake [26]. The NHC is a constant reminder for persons that they could go to the health system and request vaccines and other services. Therefore, this instrument has an important role in increasing vaccination probability. In Nigeria, immunisation cards in children increase the probability of being vaccinated against diphtheria, pertussis, and tetanus (DPT3) by 2.10 (*P* < 0.001) times [33]. Based on these findings, the NHC should be promoted as being a strategic document for older adults; in addition, health services must rigorously provide it and request it to increase VC. Additionally, health personnel need to understand the critical value that the NHC has. Healthcare professionals’ attitudes play an important role in the vaccine uptake decisions of persons [7, 10, 14, 34]. Consequently, their professional education should consider a life course perspective to promote healthy ageing with vaccination activities [7]. However, the importance of the NHC must be carefully considered, due to the different ways vaccination information was obtained in ENSANUT, directly from the NHC, or by self-reporting.

Other variables did not have the expected effect in our study, which contrasted with the evidence presented in the literature. For example, a study in the United Kingdom demonstrated that adults aged 65–92 years who were married or cohabitating with someone were 93% (*P* = 0.025, 95% CI: 1.09–3.43) more likely to be vaccinated for influenza, in contrast with those who reported being widowed, single, or divorced [35]. Additionally, a higher education level and living in urban areas increased that probability too [17]. Moreover, there is evidence that beneficiaries of specific social programs, such as *Oportunidades* (a conditional cash transfer programme to reduce poverty in Mexico), could increase VC for the full scheme in older adults (OR: 1.056, *P* < 0.001, 95% CI: 1.028–1.085) [36]. It is very likely that ENSANUT survey questions may not be able to measure this phenomenon.

On the other hand, the other two As of the taxonomy vaccine uptake model stand for awareness and acceptance. Both refer to beliefs, knowledge, safety, and efficacy perceptions of vaccines. When these variables make vaccine uptake difficult, we are referring to vaccine hesitancy. There is evidence that a higher trust in safety and efficacy alongside a high perception of contagion risk between populations could increase the probability of vaccine uptake for COVID-19 [37] and influenza [38]. It is therefore important to further investigate the role of these variables in older adults for pneumococcus, influenza, and Td vaccines.

Otherwise, the study sample is made up only of adults aged 65 and over because from that age older adults are candidates to receive the vaccines from the national vaccination programme; despite this, adults aged 60–64 with risk factors are candidates to receive the vaccine against pneumococcus, but ENSANUT does not provide information on the presence of such risk factors that could bias estimates of factors related to VC.

Finally, the reason why the 2021 ENSANUT round had fewer observations (2,025 adults aged 65 years or older who answered the age and sex questions) is that the ENSANUT was initially realised every 6 years; however, due to administrative changes, the survey started to be made annually with a small sample while maintaining national representation [39].

The main strengths of this investigation were that the estimations were made in a representative sample, as well as the fact that it considered the vaccines from the national vaccine programme, and that it is the first study to assess the factors related to VC in older Mexican adults. However, it had certain limitations. First, we calculated vaccine application estimations, and this scenario does not reflect vaccine effectivity [1]. Therefore, we cannot ensure that older people who received it were protected. The best method to evaluate effectivity is through specific antibody measurements. However, in Mexico, these data do not exist. Additionally, the measurement of antibodies in elderly individuals demonstrates another limitation in studying vaccine effectiveness. Older adults exhibit age-associated immune system deterioration changes referred to as immunosenescence. Consequently, vaccine effectiveness may be lower [40]. Second, the source of information implies a memory and information bias due to the way vaccine uptake information was obtained (from NHC or by self-reporting). Furthermore, ENSANUT did not allow us to have a more accurate analysis of the uptake dates for every vaccine. Indeed, influenza is the only vaccine that specifies the time of administration in the survey. A review of ENSANUT survey vaccine questions is desirable to better analyse vaccine coverage in older adults. Third, we could not analyse misconceptions about vaccines (such as religious dilemmas or beliefs about safety) and vaccine hesitancy with the ENSANUT, which the literature indicates as being important factors hindering vaccination uptake [10, 35]. Therefore, we did not observe the effect of the other 2As (awareness and acceptance). Fourth, although we considered the health system, we do not know the role of the physician. As has been previously stated, physician recommendations and counselling could improve vaccination uptake in older adults. Fifth, the estimates shown in this work were made considering the national representativeness of the survey, but we do not show estimates for each state or region in the country, which would be extremely important in the future to find out whether there are areas of backlog in vaccination, thereby improving the dispersion of sanitary resources in the country. Finally, it is necessary to analyse other vaccines that are available for adults, such as hepatitis A and B and herpes zoster.

In conclusion, VC in older Mexican adults was low across all years, but women had a higher percentage. The use of preventative health services, patients with morbidities, being a beneficiary of any health system institution, and the NHC property can increase vaccine uptake probability. Consequently, the Mexican health system plays a strategic role in immunisation, so it is important to increase accessibility and reduce entry barriers. Therefore, the rigorous usage of NHC and the maintenance of the current free-of-charge vaccine health policy will be essential to increase VC in older adults. Additionally, the perception of vaccine safety and its acceptance should be investigated.

## Data Availability

Data from the National Health and Nutrition Survey (Encuesta Nacional de Salud y Nutrición, ENSANUT) is available online in https://ensanut.insp.mx/.
